# Preliminary analysis of the proportions and characteristics of dual TCR T cells and dual BCR B cells in echinococcus granulosus-infected mice using scRNA+TCR+BCR-seq

**DOI:** 10.3389/fimmu.2026.1835604

**Published:** 2026-10-13

**Authors:** Peng Su, Kai Quan, Hongxia Yang, Yadong Gong, Xinsheng Yao

**Affiliations:** 1 Department of Urology, Affiliated Hospital of Zunyi Medical University, Zunyi, China; 2 Department of Immunology, Zunyi Medical University; Guizhou Provincial Research Center for Applied Molecular Immunology; Guizhou Provincial Graduate Education Innovation Base for Immunology; Guizhou Provincial Key Discipline of Immunology, Guizhou, China; 3 Central Laboratory, Guizhou Aerospace Hospital, Zunyi, China

**Keywords:** CDR3 repertoire, cystic echinococcosis, single B cell receptor sequencing, single T cell receptor sequencing, single-cell RNA sequencing

## Abstract

**Introduction:**

Cystic echinococcosis (CE) is a zoonotic parasitic disease caused by Echinococcus granulosus, and the T and B cell responses and regulatory mechanisms in CE remain unclear.

**Methods:**

In this study, we analyzed the data of peripheral blood and spleen of mice infected with Echinococcus granulosus(EG) by scRNA +TCR+BCR-seq.

**Results:**

We made the following novel findings: (1) Peripheral blood of Echinococcus granulosus (EG)-infected mice contained a higher proportion of dual TCR(T cell receptor) T cells with higher clonality than uninfected mice; (2) There was a bias in V gene usage between single TCR T and dual TCR T cells, as well as between single BCR(B cell receptor) B cells and dual BCR B cells in EG-infected mice; (3) Dual TCR Tregs, dual TCR effector CD8 T cells, dual BCR Bregs, and dual BCR memory/dividing B cells exhibited relatively high clonal expansion in EG-infected mice; (4) Shared CDR3 sequences and consistently highly expressed mRNA molecules exist between single receptor T/B and dual receptor T/B cells in EG-infected mice. KEGG enrichment analysis revealed that dual BCR B cells are closely related to the expression of factors associated with pathways such as "Th1/ Th2 differentiation" and "antigen processing and presentation".

**Discussion:**

These results suggest that dual TCR T and dual BCR B cells may be involved in the specific responses and regulation of EG-infected mice, providing a novel perspective for the adaptive immune response mechanisms and targeted therapy of parasitic infections.

## 1 Introduction

Cystic echinococcosis (CE) is one of the 17 neglected tropical diseases closely monitored by the World Health Organization, primarily caused by infection with Echinococcus granulosus, affecting over one million people. CE can damage multiple organs in the human body and is typically a lifelong disease that threatens life, making it difficult to prevent, control, and treat, resulting in annual costs exceeding 3 billion US dollars (1, 2). CE has become a global public health issue. The specific T and B cell immune responses and regulatory mechanisms in CE have not been fully elucidated. Although some progress has been made in vaccine research (3, 4), potentially effective therapeutic drugs are still awaiting clinical trials (5).

During the life cycle of Echinococcus granulosus (EG), it develops into the larval stage within the intermediate host, known as hydatid cysts. However, it does not further develop into adults within the intermediate host. These larval stages of hydatid cysts can produce eggs or larvae that can infect new hosts. The larval stage can induce an immune evasion response in the host, which helps the parasite survive long-term within the host and avoid clearance by the host’s immune system (6, 7). In the early stages of EG infection in mice, the immune response *in vivo* is predominantly Th1, but as the disease progresses to the middle and late stages, Th2 cells become dominant (8). IFN may play a protective role in Echinococcus multilocularis infection (9). In the laboratory mouse model, intraperitoneal injection of viable protoscoleces induces secondary echinococcosis, in which cystic vesicles gradually enlarge over several months and produce new protoscoleces within the host. In this model, the host immune response evolves dynamically: the early infection phase (approximately the first month) is dominated by a Th1-type response characterized by IFN-γ production, which may confer partial protection against protoscolex surviva (10); as the infection progresses to the chronic/late stage (approximately 3 months and beyond), the immune response shifts toward a Th2-dominant profile accompanied by elevated IL-4 and IL-10, along with expansion of Tregs, Bregs, and MDSCs, enabling long-term parasite survival (11). The 22-week time point selected in the present study represents this established late/chronic stage, at which the immunomodulatory microenvironment is fully developed. Treg cells proliferate during EG infection, and targeting Foxp3+ Treg cells may be effective (12). Breg cells proliferate in the late stages of EG infection and may regulate specific immune responses by modulating cytokine expression and inducing the secretion of inhibitory cytokines IL-10 and TGF-β (13).

The clonal selection theory, which posits that “a lymphocyte expresses only one type of antigen receptor,” has been challenged and supplemented by the existence of “dual TCR T and dual BCR B cells” (14–16). Since the first detection of two functional Vα and Vβ gene rearrangements in a single T cell at the mRNA level in 1988 (17, 18), multiple laboratories have identified the presence of dual TCR T and dual BCR B cells (19). In recent years, it has been confirmed that dual TCR T and dual BCR B cells are closely related to the occurrence and development of tumors, infections, and autoimmune diseases (20–27).

The antigenic epitopes and virulence factors of parasites differ from those of bacteria and viruses. To date, no studies have been reported on dual-receptor lymphocytes following parasitic infection. In this study, a detailed comparative analysis was performed on shared scRNA+TCR+BCR-seq data from EG-infected mice.In EG-infected mice, we observed high proportions and clonal expansion of double TCR T cells and double BCR B cells. Their characteristics, including CDR3 composition, originating subsets, and highly expressed mRNA molecules, suggest that these cells are closely associated with the specific immune response and regulation against EG infection. This finding provides a novel perspective for research on the adaptive immune response mechanisms and targeted therapy of parasitic infections.

## 2 Materials and methods

### 2.1 Study subjects and samples

The study subjects and samples in this study were derived from the article published by Wu J et al. (Single-Cell RNA Sequencing Reveals Unique Alterations in the Immune Panorama and Treg Subpopulations in Mice during the Late Stages of Echinococcus granulosus Infection, Infection and Immunity, Epub 2023 Apr 11, doi:10.1128/iai.00029-23). The analyzed data were obtained from the scRNA + TCR + BCR-seq sequences shared by the authors (Gene Expression Omnibus database, accession numbers: GSE216347 and GSE225312, https://www.ncbi.nlm.nih.gov/geo/query/acc.cgi?acc=GSE216347, https://www.ncbi.nlm.nih.gov/geo/query/acc.cgi?acc=GSE225312).

#### 2.1.1 Study subjects

Six to eight-week-old female BALB/c mice were randomly divided into an EG-infected group (n=3) and a non-infected control group (n=3). At 22 weeks post-infection (chronic/late infection stage), mice were euthanized by cervical dislocation, and peripheral blood and spleens were immediately collected for scRNA+TCR+BCR-seq. The detailed procedures for the preparation of infected and uninfected mice and the collection of samples can be found in the original study (Single-Cell RNA Sequencing Reveals Unique Alterations in the Immune Panorama and Treg Subpopulations in Mice during the Late Stages of Echinococcus granulosus Infection, Infection and Immunity, Epub 2023 Apr 11, doi:10.1128/iai.00029-23).

#### 2.1.2 Samples

Peripheral blood and spleens were collected from both EG-infected and non-infected mice for scRNA + TCR + BCR-seq sequencing. The samples were divided into four groups: IP = peripheral blood samples from EG-infected mice; UP = peripheral blood samples from non-infected mice; IS = spleen samples from EG-infected mice; US = spleen samples from non-infected mice. Sample names, accession numbers, total number of T and B cells sequenced, and total number of TCR and BCR pairs are shown in **Figures 1C, D**.

**Figure 1:**
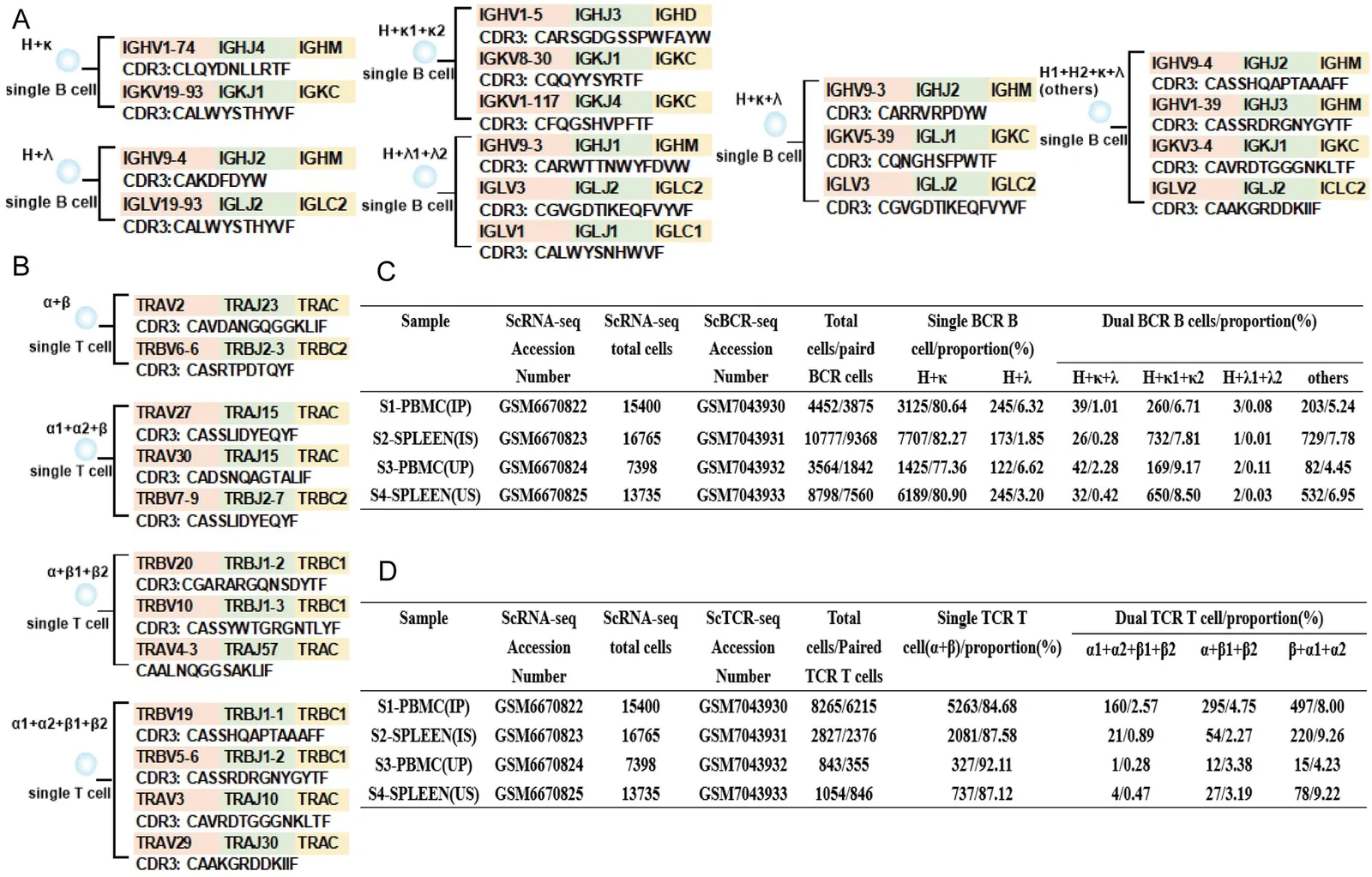
Examples of CDR3 sequences from single and dual TCR T cells and single and dual BCR B cells, along with sample names, shared data serial numbers, and counts and proportions of TCR and BCR pairing types. **(A)** Examples of CDR3 sequences for different pairing types of BCR heavy (H) chains, kappa (κ) chains, and lambda (λ) chains (H+κ; H+λ; H+κ1+κ2; H+κ+λ; H+λ1+λ2; others). **(B)** Examples of CDR3 sequences for different pairing types of TCR alpha (α) chains and beta (β) chains (α+β; α1+α2+β; α+β1+β2; α1+α2+β1+β2). **(C)** Sample names; shared serial numbers for single-cell RNA sequencing (scRNA-seq); total count of functional B cells; shared serial numbers for single-cell BCR sequencing (scBCR-seq); total count of pairable BCR B cells; counts and proportions of cells with different BCR pairing types. **(D)** Sample names; shared serial numbers for single-cell RNA sequencing (scRNA-seq); total count of functional T cells; shared serial numbers for single-cell TCR sequencing (scTCR-seq); total count of pairable TCR T cells; counts and proportions of cells with different TCR pairing types.

Infection was confirmed by gross anatomical observation of hydatid cysts in the abdominal cavity at 22 weeks post-infection, together with hematoxylin and eosin (H&E) staining of spleen sections showing characteristic pathological changes (irregularly expanded white pulp and reduced red pulp in infected mice versus normal splenic architecture in controls).

### 2.2 Analysis workflow for single BCR B cells and dual BCR B cells

scBCR-seq data analysis workflow: (1) Sequences marked as “FALSE” in the “is_cell” and “high_confidence” columns were deleted; sequences in the “Chain” column that were not “IGH,” “IGK,” or “IGL” were excluded; sequences marked as “Non” or “FALSE” in the “Productive” column were deleted; B cells with only one functional chain sequenced were removed. (2) In a single B cell, two functional chains can be paired and assembled into a single BCR B cell, referred to as BCR Heavy chain (H) + BCR light chain (Kappa or Lambda). In a single B cell, three functional chains can be paired and assembled into a dual BCR B cell, referred to as H+κ1+κ2; H+λ1+λ2; H+κ+λ. In a single B cell, four (or more) functional chains can be paired and assembled into a BCR B cell, referred to as others. Two or more BCR pairing types are collectively named dual BCR B cells. For each BCR pairing type, one B cell was selected, and the organization of the V(D)J gene family and the CDR3 amino acid sequence were listed (**Figure 1A**).

### 2.3 Analysis workflow for single TCR T cells and dual TCR T cells

scTCR-seq data analysis workflow: (1) Sequences marked as “FALSE” in the “is_cell” and “high_confidence” columns were deleted; sequences in the “Chain” column that were not “TRA” or “TRB” were excluded; sequences marked as “Non” or “FALSE” in the “Productive” column were deleted; T cells with only one functional chain sequenced were removed. (2) In a single T cell, two functional chains can be paired and assembled into a single TCR T cell, referred to as TCR alpha chain (A) + TCR beta chain (B). In a single T cell, three functional chains can be paired and assembled into a dual TCR T cell, referred to as α+β1+β2; β+α1+α2. In a single T cell, four functional chains can be paired and assembled into a multi-TCR T cell, referred to as α1+α2+β1+β2. Two or more TCR pairing types are collectively named dual TCR T cells. For each TCR pairing type, one T cell was selected, and the organization of the V(D)J gene family and the CDR3 amino acid sequence were listed (**Figure 1B**).

### 2.4 CDR3 repertoire characterization of scBCR-seq and scTCR-seq

After quality control of scTCR + BCR-seq data, the R package immunarch was used to compare and analyze the CDR3 repertoire characteristics of single TCR T, dual TCR T, single BCR B, and dual BCR B cells: (1) Clonality: The proportion of cells with clonal counts ≥2 and clonal counts =1 were compared among single TCR T, dual TCR T, single BCR B, and dual BCR B cells; (2) V gene usage bias: The usage of V gene subfamilies was compared among single TCR T, dual TCR T, single BCR B, and dual BCR B cells, with statistical analysis of V gene usage differences using SPSS; (3) Overlap: The overlap of CDR3 sequences was analyzed using the R package immunarch; (4) Each T and B cell’s unique barcode was used to integrate scRNA-seq and scTCR + BCR-seq data for analysis.

### 2.5 scRNA-seq data quality control and integration with scTCR + BCR-seq

The scRNA-seq data shared in GEO were imported into R for analysis: (1) The harmonyR package was used for sample integration, and cells with more than 12,000 or fewer than 200 unique genes were excluded from the analysis; (2) Cells with mitochondrial gene percentages exceeding 5% and log10GenesPerUMI (genes per UMI) less than 0.8 were removed from the dataset, and only genes expressed in 10 or more cells were used for further analysis; (3) Doublets were identified and removed using the “DoubletFinder” R package in conjunction with the “Seurat” R package version 5; (4) Normalization was performed with the normalization method LogNormalize in the Seurat package (version 4.0.1) and with a scale factor with a default value of 10,000; (5) The Seurat object’s ScaleData, FindVariableFeatures, and RunPCA functions were analyzed, and dimensionality reduction clustering was performed using the RunUMAP function; the top 10 mRNA expressions of each sample subgroup were selected using the FindAllMarkers function; the “SingleR” package was used to annotate cell types, and the clustering results were corrected based on the expression of canonical genes; (6) T cells were clustered based on the expression of canonical markers and cell subgroups: EffectorCD8T (Cd8a, Gzmk, Emoes, Ly6c1, Ly6c2, Nkg7); NaiveCD8T (Cd8a, Sell, Ccr7); NaiveCD4T (Cd4, Sell, Ccr7); IFN_CD4 (Cd4, Isg15); Th1 (Cd4, Tbx21, Stat4, Ifr8); Th2 (Cd4, Stat6, Il-4); Treg (Cd4, Foxp3); (7) B cells were clustered based on the expression of canonical markers and cell subgroups: Naive_B (Cd19, Ighd); Memory_B (Cd19, Ighd, Cd27); Breg (Cd19, Cd1d1, Cd1d2); Plasma (Cd19, Jchain, Xbp1); Dividing_B (Cd19, Mki67, H2afz, Cdk1, Aicda); (8) Data that met both scRNA-seq quality control and scTCR + BCR-seq screening criteria were integrated for analysis based on each cell’s unique barcode.

### 2.6 Major tools, software, and statistical methods

Software packages: The R packages “Seurat,” “ggplot2,” “DoubletFinder,” Adobe, and GraphPad Prism (version 5) were used for data visualization; data analysis was performed using R studio (v3.3.3) and GraphPad Prism (version 5); the “SingleR” package was used for cell type annotation, with manual correction based on canonical gene expression; Statistical methods: IBM SPSS Statistics 26 software was used for statistical analysis; when n ≥ 40 and Tmin ≥ 5, Pearson’s chi-square test was used to compare multiple ratios between two groups; when n < 40 or Tmin < 1, Fisher’s exact test was used.(*P>0.05).

## 3 Result

### 3.1 Proportion and pairing characteristics of dual TCR T and dual BCR B cells

Dual TCR T and dual BCR B cells were identified in the spleen and peripheral blood of EG-infected mice, with CDR3 sequences shown in **Figures 1A, B**. The proportions of dual BCR B cells in the peripheral blood and spleen of EG-infected mice were 13.03% and 15.88%, respectively, with the main pairing type being H + K1 + K2 (6.71% in peripheral blood; 7.81% in spleen) (**Figure 1C**). The proportions of dual TCR T cells in the peripheral blood and spleen of EG-infected mice were 15.32% and 12.42%, respectively, with the main pairing type being A1 + A2 + B (8.00% in peripheral blood; 9.26% in spleen) (**Figure 1D**).

#### 3.1.1 Dual TCR T cells

IP group (15.32%) > UP group (7.89%); no difference between US (12.88%) and IS groups (12.42%) (**Figure 2A**); IP group (15.32%) > IS group (12.42%) (**Figure 2C**).

**Figure 2:**
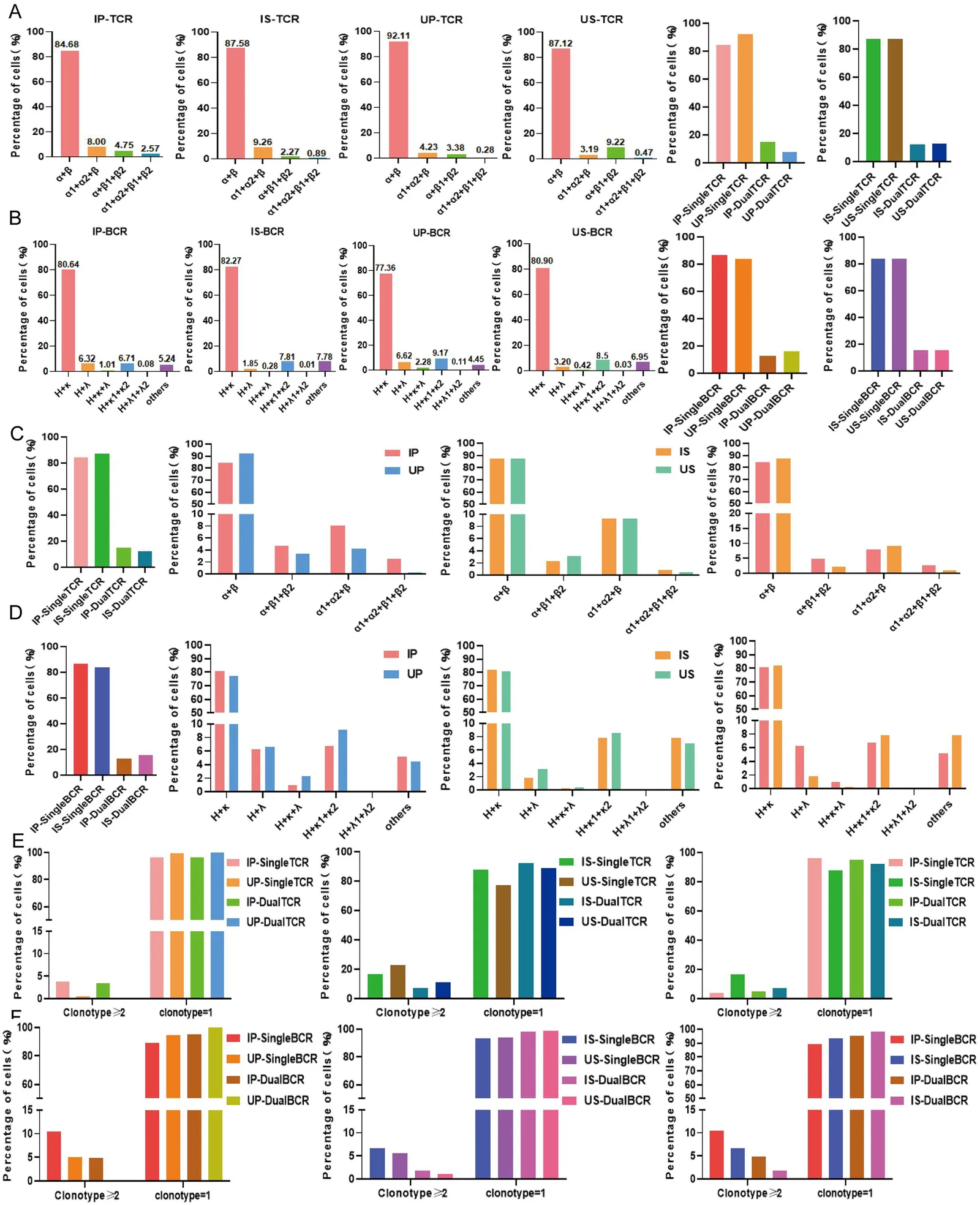
Analysis of TCR and BCR CDR3 pairing types and clonality in IP, IS, UP, and US using scTCR-seq and scBCR-seq. **(A)** Proportions of single TCR T and dual TCR T pairing types in IP, IS, UP, and US, with comparisons between total single TCR T and total dual TCR T (IP vs. UP; IS vs. US). **(B)** Proportions of single BCR B and dual BCR B pairing types in IP, IS, UP, and US, with comparisons between total single BCR B and total dual BCR B (IP vs. UP; IS vs. US). **(C)** Comparison of the proportions of total single TCR T and total dual TCR T cells between the IP and IS groups; comparison of TCR pairing types among IP, IS, UP, and US groups. **(D)** Comparison of the proportions of total single BCR B and total dual BCR B cells between the IP and IS groups; comparison of BCR pairing types among IP, IS, UP, and US groups. **(E)** Comparison of clonal expansion (clonotype ≥ 2) of single TCR T and dual TCR T cells among IP, IS, UP, and US. **(F)** Comparison of clonal expansion (clonotype ≥ 2) of single BCR B and dual BCR B cells among IP, IS, UP, and US.

#### 3.1.2 Dual BCR B cells

UP group (16.02%) > IP group (13.03%); no difference between US group (15.90%) and IS group (15.88%) (**Figure 2B**); IS group (15.88%) > IP group (13.03%) (**Figure 2D**).

Among the four groups, the dual TCR T cell pairing type with the highest proportion was A1 + A2 + B, in the order of IS (9.26%) > US (9.22%) > IP (8.00%) > UP (4.23%). For dual BCR B cells, the most common pairing type was H + K1 + K2, with proportions in the order of UP (9.17%) > US (8.50%) > IS (7.81%) > IP (6.71%) (**Figures 2C, D**).

### 3.2 Clonality of CDR3 repertoire

Clonal expansion (clonetype ≥2) proportions were compared as follows: (1) IP single TCR (3.84%) > IP dual TCR (3.47%) > UP single TCR (0.62%) > UP dual TCR (0%); US single TCR (22.93%) > IS single TCR (16.97%) > US dual TCR (11.01%) > IS dual TCR (7.46%); IS single TCR (16.97%) > IS dual TCR (7.46%) > IP single TCR (3.84%) > IP dual TCR (3.47%) (**Figure 2E**). (2) IP single BCR (10.53%) > UP single BCR (5.11%) > IP dual BCR (4.95%) > UP dual BCR (0%); IS single BCR (6.70%) > US single BCR (5.70%) > IS dual BCR (1.75%) > US dual BCR (1.16%); IP single BCR (10.53%) > IS single BCR (6.70%) > IP dual BCR (4.95%) > IS dual BCR (1.75%) (**Figure 2F**).

### 3.3 V gene usage in CDR3 repertoire

In the IP group, dual TCR T cells preferentially used TRAV3 and TRBV17, while single TCR T cells preferentially used TRAV5 and TRBV2. Dual BCR B cells preferentially used IGHV8, while single BCR B cells preferentially used IGHV1 (**Supplementary Figure 1A**). In the IS group, dual TCR T cells preferentially used TRAV1 and TRBV10, while single TCR T cells preferentially used TRAV11. Single BCR B cells preferentially used IGHV9 (**Supplementary Figure 1B**).

Compared to the UP group, dual TCR T cells in the IP group preferentially used TRBV19. Compared to the US group, dual TCR T cells in the IS group preferentially used TRBV12. Dual BCR B cells preferentially used IGHV8 and IGHV14. When comparing the IP and IS groups, dual TCR T cells in the IP group preferentially used TRBV5, while dual TCR T cells in the IS group preferentially used TRAV1 and TRBV2. No significant differences were observed in V gene usage between dual BCR B cells in the IP and IS groups (**Supplementary Figures 1C–E**).

### 3.4 Dual TCR T and dual BCR B cells in mouse peripheral blood samples

T cell subset clustering in EG-infected mice: Effector CD8T, IFN CD4T, Naïve CD4T, Naïve CD8T, Th1, Th2, Treg (**Figure 3A**). Dual TCR T cells were identified in every subset. The subset distribution of dual TCR T cells was as follows: Naïve CD4T (72.77%) > Naïve CD8T (12.90%) > Treg (5.29%) > IFN CD4T (4.77%) > Effector CD8T (3.10%) > Th2 (0.90%) > Th1 (0.26%). Regarding clonal expansion among T cell subsets, dual TCR T cells exhibited clonal expansion only in Effector CD8T, Naïve CD4T, and Naïve CD8T, with Effector CD8T showing the highest level. For single TCR T cells, Effector CD8T also displayed the highest clonal expansion (**Figure 3B**).

**Figure 3:**
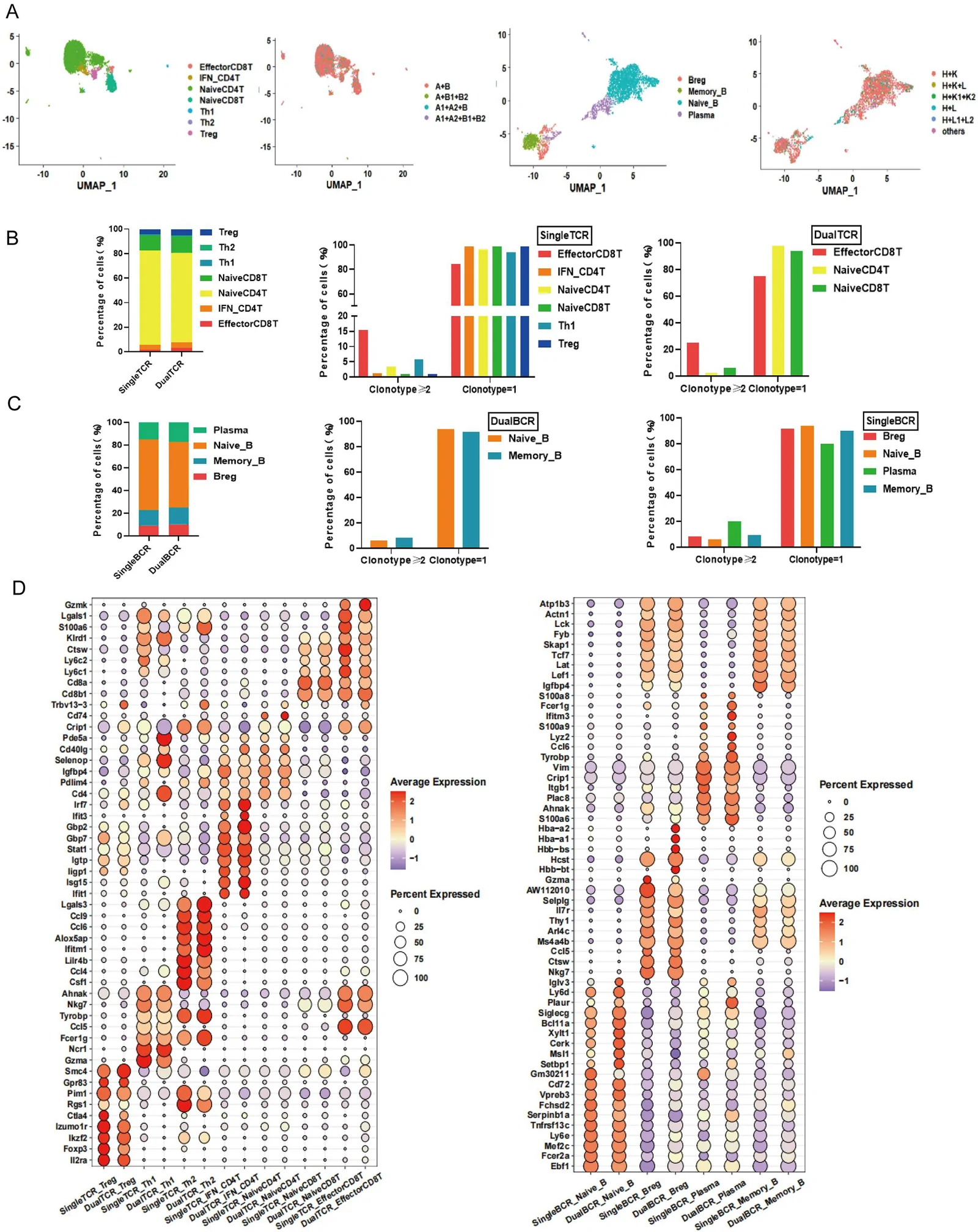
Analysis of T and B cell subsets, clonality, and mRNA expression in the IP group using ScRNA+TCR+BCR-seq. **(A)** UMAP clustering maps of T cell and B cell subsets, with distribution charts of TCR and BCR pairing types. **(B)** Comparison of the distribution and clonal expansion (clonotype ≥ 2) of single TCR T cell and dual TCR T cell subsets. **(C)** Comparison of the distribution and clonal expansion (clonotype ≥ 2) of single BCR B cell and dual BCR B cell subsets. **(D)** Comparison of the top 10 mRNA expressions in single TCR T and dual TCR T cells, and comparison of the top 10 mRNA expressions in single BCR B and dual BCR B cells.

B cell subset clustering in EG-infected mice: Breg, Plasma, Naïve_B, Memory B (**Figure 3A**). Double BCR B cells were present in every subset. The subset distribution of double BCR B cells was: Naïve B (57.73%) > Plasma (17.11%) > Memory B (14.85%) > Breg (10.31%). Regarding clonal expansion among B cell subsets, single BCR B cells showed the highest clonal expansion in Plasma cells, whereas double BCR B cells exhibited clonal expansion only in Naïve B and Memory B, with no significant difference between these two subsets (**Figure 3C**).

mRNA expression analysis between single TCR T cells and dual TCR T cells within each T cell subset: The mRNA expression of effector molecules was generally similar between single TCR T and dual TCR T cells across subsets. However, some T cell subsets showed higher expression of characteristic genes in dual TCR T cells. For example, dual TCR Th2 cells expressed higher IL-4 than single TCR Th2 cells; dual TCR IFN CD4T cells expressed higher Isg15, Isg20, and Ifit3 than single IFN CD4T cells (**Supplementary Figure 2A**). mRNA expression analysis between single BCR B cells and double BCR B cells within each B cell subset: The mRNA expression of effector molecules was generally similar between single BCR B and double BCR B cells across subsets. Nevertheless, some B cell subsets exhibited higher expression of characteristic genes in double BCR B cells. For instance, double BCR Breg cells expressed higher Cd1d1 than single BCR Breg cells (**Supplementary Figure 2A**).

Uninfected mice: The subset distribution of dual TCR T cells included Naïve CD4T, Naïve CD8T, Treg, and Effector CD8T, with no clonal expansion of dual TCR T cells detected. The subset distribution of double BCR B cells included Plasma, Naïve B, and Memory B, with no clonal expansion of double BCR B cells observed (**Supplementary Figures 4A–C**).

### 3.5 Dual TCR T and dual BCR B cells in spleen samples

EG-infected mice: T cell subsets were clustered as Effector CD8T, IFN CD4T, Naïve CD4T, Naïve CD8T, and Th2 (**Figure 4A**). Dual TCR T cells were present in every T cell subset. The subset distribution of dual TCR T cells was: Naïve CD4T (36.99%) > Treg (21.92%) > Naïve CD8T (17.35%) > IFN CD4T (12.79%) > Effector CD8T (6.85%) > Th2 (4.11%). Regarding clonal expansion among T cell subsets, dual TCR T cells showed clonal expansion only in Effector CD8T, IFN CD4T, and Treg, with Effector CD8T displaying the highest level. For single TCR T cells, clonal expansion was mainly observed in Effector CD8T and IFN CD4T (**Figure 4B**). B cell subsets were clustered as Breg, Plasma, Naïve B, Memory B, and Dividing B (**Figure 4A**). Double BCR B cells were present in every B cell subset. The subset distribution of double BCR B cells was: Naïve B (73.44%) > Breg (13.74%) > Dividing B (8.26%) > Plasma (3.99%) > Memory B (0.56%). For clonal expansion among B cell subsets, single BCR B cells showed the highest clonal expansion in Plasma cells, whereas double BCR B cells with clonal expansion were mainly Dividing B cells (**Figure 4C**).

**Figure 4:**
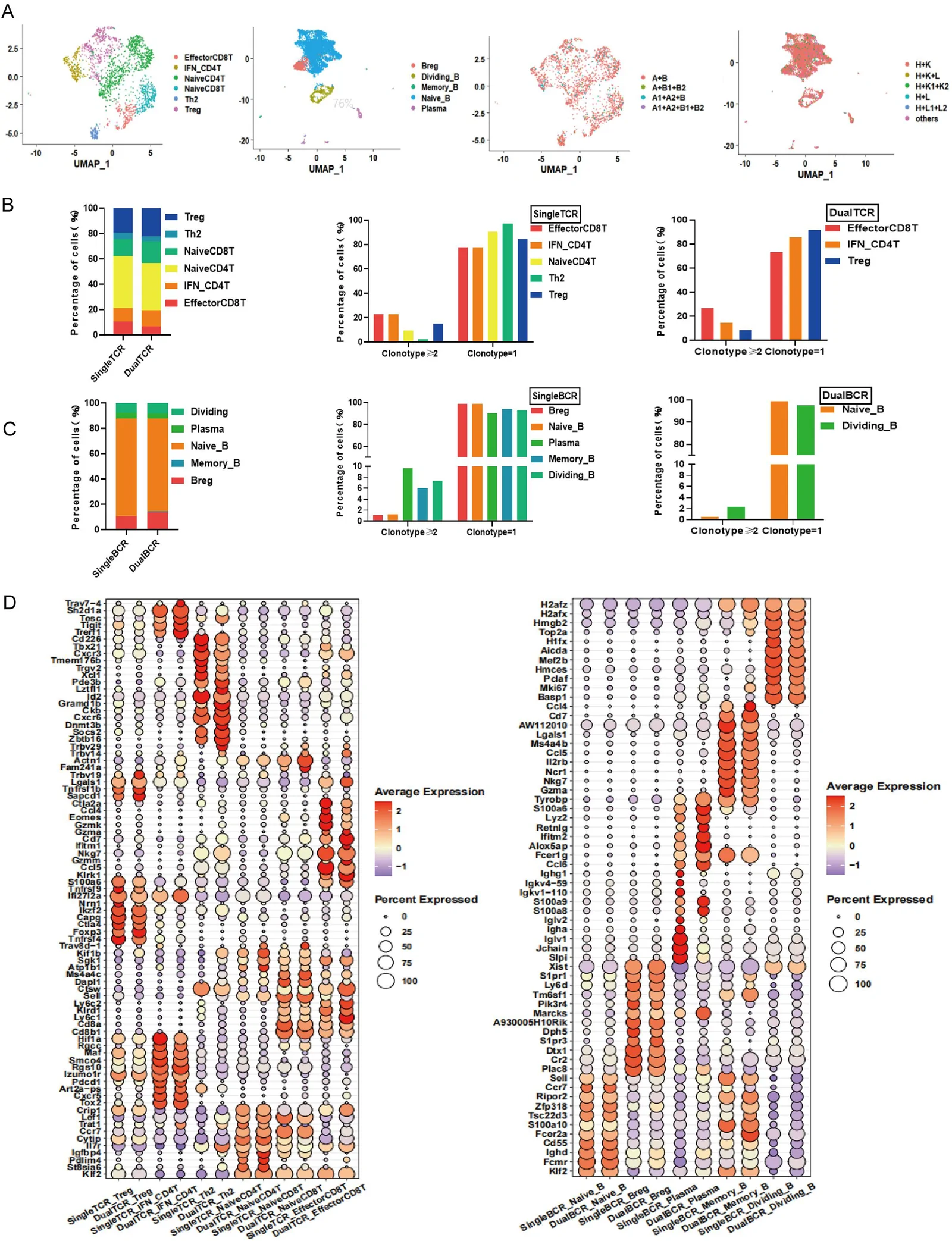
Analysis of T and B cell subsets, clonality, and mRNA expression in the IS group using ScRNA+TCR+BCR-seq. **(A)** UMAP clustering plots of T cell and B cell subsets; distribution plots of TCR and BCR pairing types. **(B)** Comparison of the distribution and clonal expansion (clonotype ≥ 2) among single TCR T cell and dual TCR T cell subsets. **(C)** Comparison of the distribution and clonal expansion (clonotype ≥ 2) among single BCR B cell and dual BCR B cell subsets. **(D)** Comparison of the top 10 mRNA expressions in single TCR T and dual TCR T cells, and the top 10 mRNA expressions in single BCR B and dual BCR B cells.

Within each T cell subset, the mRNA expression of effector molecules was generally similar between single TCR T and dual TCR T cells, but some subsets exhibited higher expression of characteristic genes in dual TCR T cells. For example, dual TCR Treg cells expressed higher Foxp3 than single TCR Treg cells (**Supplementary Figure 3A**). Within each B cell subset, the mRNA expression of effector molecules was generally similar between single BCR B and double BCR B cells (**Supplementary Figure 3A**).

Uninfected mice: Among dual TCR T subsets (Naïve CD4T, Naïve CD8T, Treg, Effector CD8T), clonal expansion was mainly observed in dual TCR Naïve CD4T cells. Among double BCR B subsets (Plasma, Naïve B, Memory B), only double BCR Naïve B cells showed clonal expansion (**Supplementary Figures 5A–C**).

### 3.6 Comparative analysis of top 10 mRNAs and shared CDR3s between single TCR T (or single BCR B) and dual TCR T (or double BCR B) cells, and KEGG comparison of total dual TCR T and total double BCR B cells

EG-infected mice: Among each single TCR T and dual TCR T subset, as well as each single BCR B and double BCR B subset, the expression profiles of the top 10 mRNAs were generally consistent (**Figures 3D**, **4D**).

Uninfected mice: The consistency of the top 10 mRNA expression between each single TCR T and dual TCR T subset, and between each single BCR B and double BCR B subset, was lower than that observed in EG-infected mice (**Supplementary Figures 4D**, **5D**).

Shared CDR3 sequences in EG-infected and uninfected mice: Partial sharing of CDR3 sequences was observed among single TCR T cell subsets, among dual TCR T cell subsets, and between single and dual TCR T cell subsets. Similarly, partial sharing of CDR3 sequences was observed among single BCR B cell subsets, among double BCR B cell subsets, and between single and double BCR B cell subsets. The number of shared CDR3s was higher in EG-infected mice than in uninfected mice (**Supplementary Figures 2B**, **3B**, **4E**, **5E**).

Comparison between spleen and peripheral blood in EG-infected and uninfected mice: Differences in the top 10 mRNAs of total dual TCR T cells and total double BCR B cells were observed between the spleen and peripheral blood (**Supplementary Figures 6A, B**).

KEGG enrichment analysis of total dual TCR T and total double BCR B cells in EG-infected mice: In peripheral blood, both single BCR B and double BCR B cells showed significant enrichment of gene sets such as “Th1 and Th2 differentiation” and “Antigen processing and presentation”. In the spleen, both single BCR B and double BCR B cells showed significant enrichment of gene sets such as “Intestinal immune network for IgA production” and “Antigen processing and presentation” (**Supplementary Figures 7A–D**, **8A–D**).

## 4 Discussion

The specificity, diversity, and memory of T and B cell responses to antigens are based on the clonal selection theory, which posits that “a lymphocyte expresses only one type of antigen receptor.” (28) However, incomplete V(D)J allelic exclusion recombination can lead to the existence of “dual receptor lymphocytes.” In recent years, dual TCR T and dual BCR B cells have been shown to be closely related to the occurrence and development of tumors, infections, and autoimmune diseases (14, 19–27, 29–31). Whether dual TCR T and dual BCR B cells are involved in the immune response to parasitic infections has not been reported. In this study, we leveraged the disruptive advantages of scRNA + TCR + BCR-seq technology in the analysis of dual TCR T and dual BCR B cells to conduct a detailed comparative analysis of the single-cell study data on EG-infected mice by Wu J et al. We innovatively found that dual TCR T and dual BCR B cells may be involved in the immune response and regulation of EG-infected mice, providing a novel perspective for the adaptive immune response mechanisms and new therapeutic targets in parasitic infections.

### 4.1 Proportion analysis of dual receptor lymphocytes

The proportion of dual TCR T cells in peripheral blood was higher in EG-infected mice than in uninfected mice, whereas in the spleen, the proportions were similar between the two groups, suggesting that dual TCR T cells in peripheral blood are more closely associated with EG infection. In uninfected mice, the proportion of double BCR B cells in peripheral blood was higher than that in EG-infected mice, indicating that single BCR B cells are the main B-cell population involved in peripheral blood during EG infection.

### 4.2 Clonal expansion analysis of dual receptor lymphocytes

The clonal expansion of both dual TCR T cells and double BCR B cells in peripheral blood was higher in EG-infected mice than in uninfected mice. In the spleen, clonal expansion of double BCR B cells was also higher in EG-infected mice than in uninfected mice, suggesting that dual-receptor lymphocytes may participate in the immune response or regulation against EG infection. However, in the spleen, clonal expansion of dual TCR T cells was lower in EG-infected mice compared to uninfected mice. Further comparison of dual TCR Treg cells in the spleen between EG-infected and uninfected mice revealed that the expression of inhibitory factors, including PDCD1, Lag3, and CTLA4, was significantly higher in dual TCR Treg cells from EG-infected mice, indicating that dual TCR Treg cells exerting suppressive effects may be closely associated with EG infection (**Supplementary Figure 3A**) (32–34).

### 4.3 Analysis of V gene family usage in dual-receptor lymphocytes

Between EG-infected and uninfected mice, dual TCR T cells showed biased usage of multiple TRAV and TRBV subfamilies, and double BCR B cells showed biased usage of multiple IGHV subfamilies, suggesting that dual TCR T cells and double BCR B cells may participate in immune responses against different antigenic epitopes of EG. Additionally, differences in V gene usage were also observed between the spleen and peripheral blood of EG-infected mice, indicating that the antigen recognition sequences of dual TCR T cells in the spleen and peripheral blood may exhibit tissue specificity (**Supplementary Figures 1C–E**).

### 4.4 Subset distribution of dual-receptor lymphocytes

In the peripheral blood of EG-infected mice, dual TCR T cells were distributed across multiple T cell subsets, including Treg, Th1, Th2, naïve CD4T, naïve CD8T, IFN CD4T, and effector CD8T. Among these, dual TCR effector CD8+ T cells exhibited the highest proportion of clonal expansion (clonotype ≥2). In the spleen of EG-infected mice, dual TCR T cells were derived from various subsets but lacked Th1 cells. Among splenic subsets, dual TCR effector CD8+ T cells, dual TCR Treg cells, and dual TCR IFN CD4+ T cells showed relatively high proportions of clonal expansion. In contrast, in uninfected mice, clonal expansion was mainly observed in dual TCR naïve CD4+ T cells (**Supplementary Figures 4A, B**, **5A, B**), suggesting that dual TCR effector CD8+ T cells are closely associated with EG-infected mice; In our study of infected mice, we found that the expression profiles of characteristic genes and cytokines were generally similar between single TCR T cells and dual TCR T cells (**Supplementary Figures 2A**, **3A**), indicating that single TCR T and dual TCR T cells exert similar immune effector functions. Treg cells, through the expression of inhibitory transcription factors such as Foxp3, play important roles in tumor immunity, autoimmunity, and immune responses to parasitic infections (35–38). Therefore, the study of dual TCR Treg cells has consistently attracted widespread attention (39, 40). In our previous studies, we identified potentially functional dual TCR Treg cells in patients with ankylosing spondylitis (AS) and Kawasaki disease (KD) (40, 41).In the present study, we found that the proportion of dual TCR Treg cells in both peripheral blood and the spleen was higher than that of single TCR Treg cells (**Figures 3B**, **4B**). Further analysis revealed that, in the spleen of EG-infected mice, dual TCR Treg cells expressed higher levels of inhibitory genes such as Foxp3 and Ikzf2 compared to single TCR Treg cells, suggesting that splenic dual TCR Treg cells may exert stronger immune regulation and represent an important direction for further research on parasitic infections.

In the early stages of Echinococcus granulosus infection, the immune response is primarily dominated by Th1 cells, which regulate the immune response through transcription factors such as Tbx21 and Stat4, leading to the production of specific cytokines like IFN-γ (42, 43). In the peripheral blood of EG-infected mice, we observed higher expression of Tbx21 and Stat4 in single TCR Th1 cells compared to dual TCR Th1 cells (**Supplementary Figure 2A**), suggesting that single TCR Th1 cells may play a primary role in the immune response. As EG infection progresses, the immune response shifts to a Th2-dominated response, which primarily involves cells and humoral immunity (44). Th2 cells mainly secrete IL-4, which promotes Th2-type immune responses, inhibits Th1-type immune responses, and regulates the balance between cell-mediated and humoral immune responses, particularly in combating multicellular parasitic infections such as helminths (45–48). For example, IL-4 stimulates B cell proliferation and differentiation into antibody-producing cells, particularly increasing the production of IgE and IgG1 (49). In the peripheral blood and spleen of EG-infected mice, we found that single TCR Th2 cells exhibited significantly higher expression of IL-4 compared to dual TCR Th2 cells, suggesting that single TCR Th2 cells play a primary role in immune regulation. High expression of Gata3 in Th2 cells inhibits the production of Th1 cells (50, 51). In the peripheral blood of EG-infected mice, we observed low expression of Gata3 in dual TCR Th2 cells, while Th1 cell clusters were identified. This suggests that the inhibitory effect of dual TCR Th2 cells may be limited. In contrast, in the spleen of EG-infected mice, dual TCR Th2 cells exhibited high expression of Gata3, and no Th1 cell clusters were identified. This suggests that dual TCR Th2 cells in the spleen may influence Th1 cell differentiation through high expression of Gata3.

In EG-infected mice, double BCR B cells in peripheral blood were derived from multiple B cell subsets, including Breg, naïve B, memory B, and plasma cells, with clonal expansion of double BCR memory B cells being higher than that of double BCR naïve B cells. In the spleen of EG-infected mice, double BCR B cells additionally included double BCR dividing B cells (which were not clustered in peripheral blood), and clonal expansion of double BCR dividing B cells was higher than that of double BCR naïve B cells. In uninfected mice, clonal expansion was mainly observed in double BCR naïve B cells(**Supplementary Figures 4A, C**, **5A, C**). The high clonal expansion rates of double BCR memory B cells and double BCR dividing B cells in EG-infected mice suggest their close involvement in the humoral immune response against EG infection. In clonal expansion analyses of B cell subsets from peripheral blood and spleen of EG-infected mice, we found that single BCR plasma cells exhibited the highest clonal expansion in both spleen and peripheral blood samples, indicating that single BCR plasma cells are the dominant B cell subset in the immune response to parasitic infection (13). In the gene expression analysis of infected mice, we found that the expression profiles of characteristic genes in single BCR B and double BCR B cells were largely consistent (**Supplementary Figures 2A**, **3A**), suggesting that single BCR B and double BCR B cells may exert similar immune effector functions.

Breg cells are a subset of B cells with immunomodulatory functions. They participate in immune regulatory responses in various diseases such as autoimmune diseases, infections, and tumors. Breg cells can suppress inflammatory responses by secreting anti-inflammatory cytokines including IL-10 and TGF-β (52).In the peripheral blood and spleen of EG-infected mice, we found that the proportion of double BCR Breg cells was higher than that of single BCR Breg cells (**Figures 3C**, **4C**). Moreover, the proportion of double BCR Breg cells in the spleen was higher than that in peripheral blood. Further analysis revealed that double BCR Breg cells in peripheral blood expressed higher levels of Cd1d1 compared to single BCR Breg cells (**Supplementary Figure 2A**). It is known that Breg cells present lipids via CD1d and induce iNKT cells to differentiate into a novel iNKT cell subset with anti-inflammatory functions (53).This suggests that, in the peripheral blood immune environment of EG-infected mice, double BCR Breg cells represent an important direction for future research on parasitic infections.

KEGG enrichment analysis was performed on single BCR B and double BCR B cells from peripheral blood and spleen samples of EG-infected mice. In peripheral blood, both single BCR B and double BCR B cells showed significant enrichment of gene sets such as “Th1 and Th2 differentiation” and “Antigen processing and presentation”. In the spleen, both single BCR B and double BCR B cells showed significant enrichment of gene sets such as “Intestinal immune network for IgA production” and “Antigen processing and presentation”. These findings suggest that double BCR B cells may play an important regulatory role in the specific immune responses of T and B cells during EG infection (**Supplementary Figures 7**, **8**).

A high degree of CDR3 overlap was observed between single TCR T and dual TCR T cells, as well as between single BCR B and double BCR B cells, in EG-infected mice (**Supplementary Figures 2E**, **3E**). In contrast, very little CDR3 overlap was detected in the corresponding samples from uninfected mice (**Supplementary Figures 4E**, **5E**). These findings suggest that dual TCR T (or double BCR B) cells and their single-receptor counterparts respond to the same antigenic epitopes and are associated with the specific immune response to EG infection. This is consistent with our previous findings in autoimmune disease (AS), infectious disease (KD), and tumor (lung cancer), where shared CDR3 sequences were also observed between single TCR T and dual TCR T cells (41, 42, 54).

Further global comparison of the top 10 mRNA expression profiles between single TCR T and dual TCR T cells within each subset, as well as between single BCR B and double BCR B cells within each subset (**Figures 3D**, **4D**), revealed that they were largely consistent, suggesting that dual-receptor T or B cells exert immune effector functions similar to those of single-receptor T or B cells. At the same time, certain differences were observed in the expression of some cytokines, cytokine receptors, and transcription factors between single and dual cells within each subset, indicating potential differences in the magnitude of immune response and regulation between them. However, it should be emphasized that this study is only a comparison at the mRNA level between EG-infected and uninfected mice, and the observed proportions and characteristics of dual TCR T and double BCR B cells need further validation at the protein expression level.

Through a detailed comparative analysis of shared scRNA+TCR+BCR-seq data from the spleen and peripheral blood of EG-infected and uninfected mice, we report for the first time that dual TCR T cells and double BCR B cells in EG-infected mice may be associated with the specific immune response and regulation against EG infection, particularly the clonally expanded dual TCR Treg, dual TCR effector CD8 T, double BCR Breg, and double BCR memory/dividing B cells. These findings provide novel research directions for understanding the complex T and B cell responses following parasitic infection. Nevertheless, it is important to emphasize that although studies on the protein expression levels and functional effects of dual-receptor lymphocytes have been confirmed and widely applied under physiological and various pathological conditions (54–58). The findings of this study warrant further investigation using tools such as fluorescent gene reporter mice to establish an EG parasitic infection model, thereby enabling in-depth studies on the potential specific immune response mechanisms and effector functions of dual-receptor lymphocytes.

## Data Availability

The data used in this study are all sourced from public databases (Gene Expression Omnibus repository). scRNA-seq, scTCR-seq, and scBCR-seq data can be found in the Gene Expression Omnibus repository, accession numbers GSE216347 and GSE225312. The data in this database are publicly available under open-access terms, and their use does not require authorization or approval from any institution or regulatory body. The datasets generated and/or analyzed during this study are available from the corresponding author on reasonable request.
