# Supplementary material for: Preliminary analysis of the proportions and characteristics of dual TCR T cells and dual BCR B cells in echinococcus granulosus-infected mice using scRNA+TCR+BCR-seq
Source: Front Immunol. 2026 Oct 13;17:1835604. doi: 10.3389/fimmu.2026.1835604 (PMC13710079; doi:10.3389/fimmu.2026.1835604)
Supplement: Supplementary file 1 [file DataSheet1.pdf]

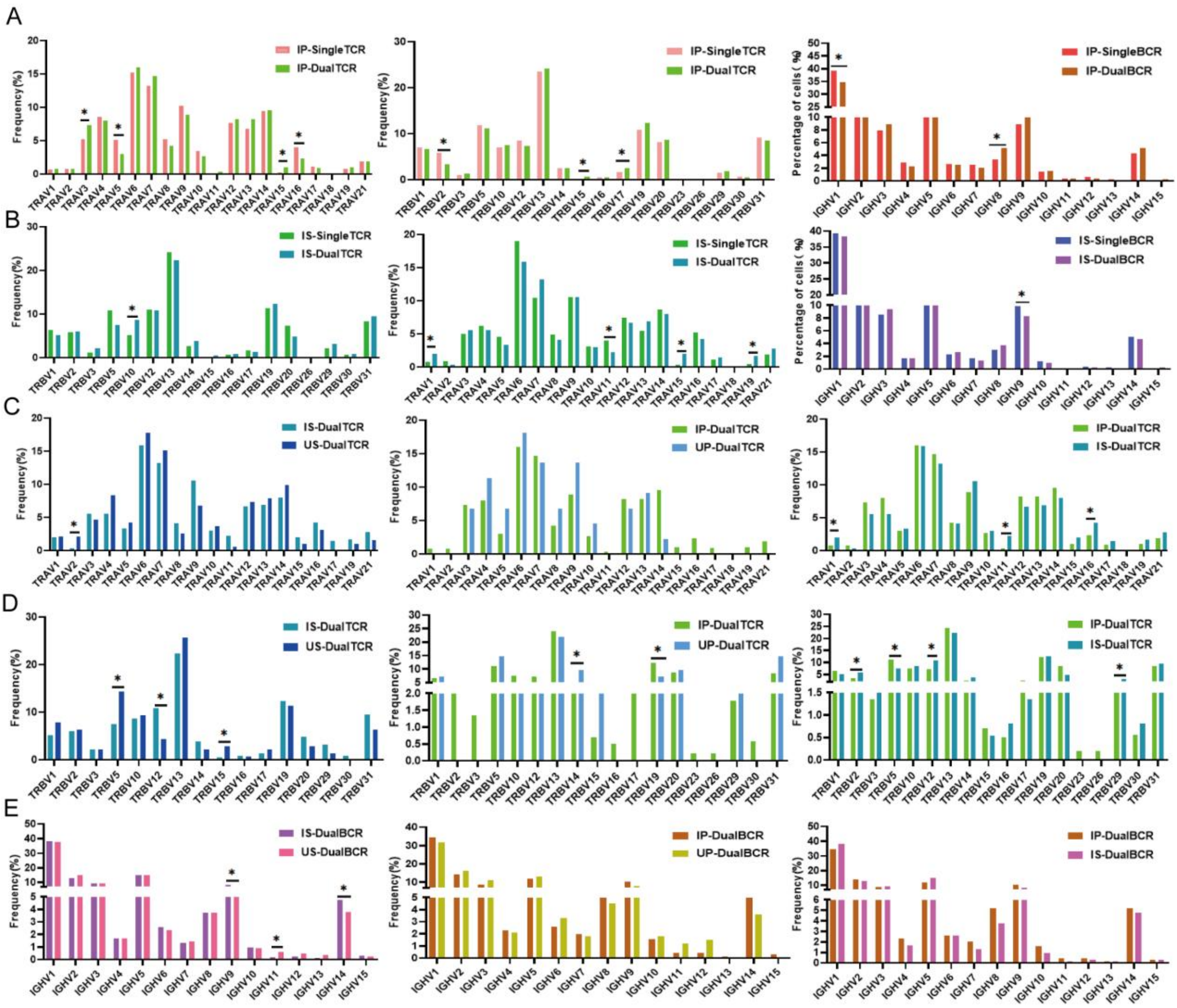

Supplementary Figure 1. Analysis of TRAV, TRBV, and IGHV subfamily usage in single TCR T, dual TCR T, single BCR B, and dual BCR B cells from four groups of samples using scTCR+TCR+BCR-seq.

A. Proportions of TRAV and TRBV subfamily usage in single TCR T cells and dual TCR T cells of the IP group; proportions of IGHV subfamily usage in single BCR B cells and dual BCR B cells of the IP group.

B. Proportions of TRAV and TRBV subfamily usage in single TCR T cells and dual TCR T cells of the IS group; proportions of IGHV subfamily usage in single BCR B cells and dual BCR B cells of the IS group.

C. Analysis of TRAV subfamily usage proportions across different samples (IP, UP, IS, US).

D. Analysis of TRBV subfamily usage proportions across different samples (IP, UP, IS, US).

E. Analysis of IGHV subfamily usage proportions across different samples (IP, UP, IS, US). (Pearson's chi-square test, *$P > 0.05$)

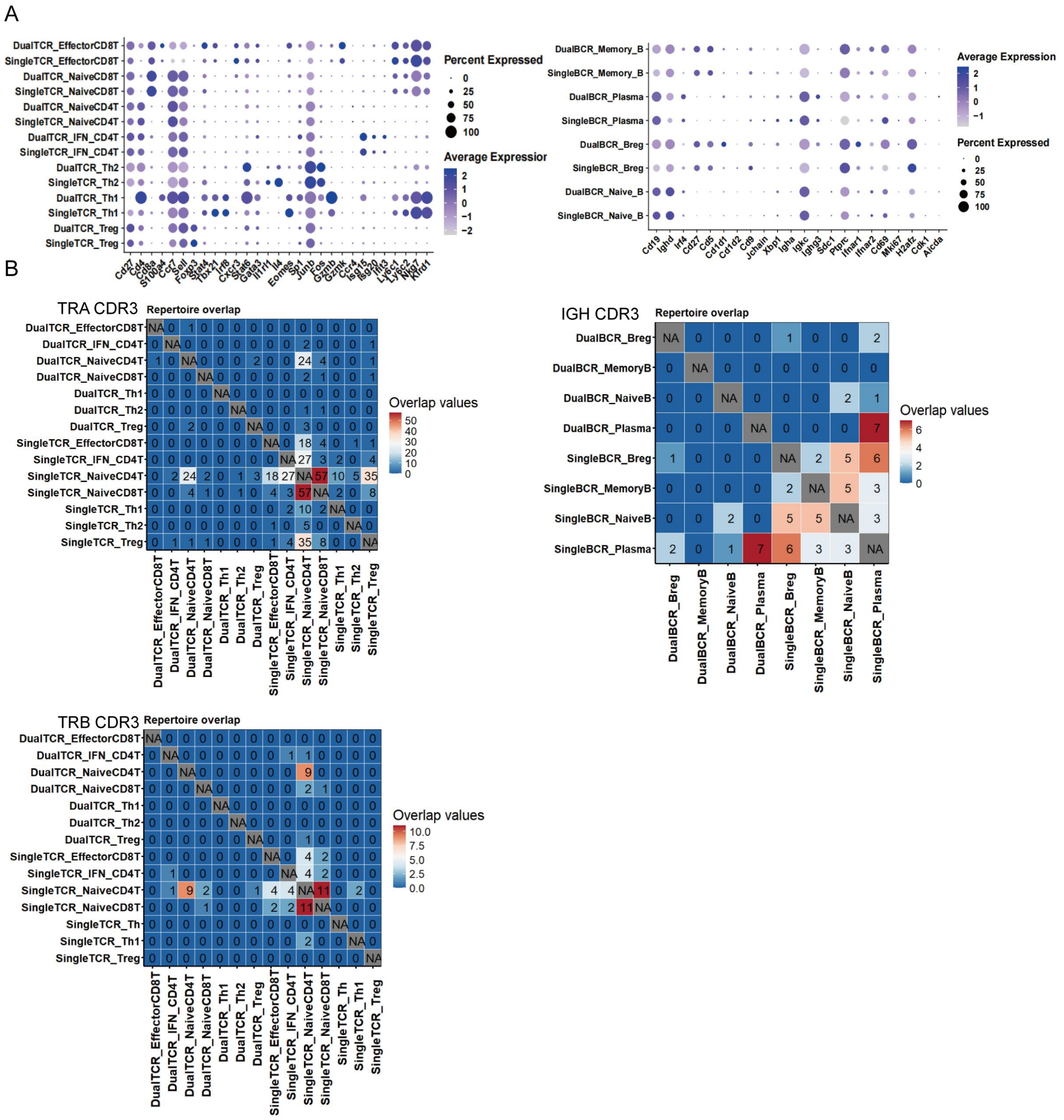

Supplementary Figure 2. Analysis of characteristic transcriptional genes and CDR3 overlaps in single TCR T, dual TCR T, single BCR B, and dual BCR B cell subsets of the IP group using scTCR+TCR+BCR-seq.

A. Analysis of characteristic gene and cytokine expression in single TCR T and dual TCR T cell subsets of the IP group; analysis of characteristic gene and cytokine expression in single BCR B and dual BCR B cell subsets of the IP group.

B. Number of overlapping TRA CDR3 AA sequences in single TCR T and dual TCR T cell subsets of the IP group; number of overlapping TRB CDR3 AA sequences in single TCR T and dual TCR T cell subsets; number of overlapping IGH CDR3 AA sequences in single BCR B and dual BCR B cell subsets.

A

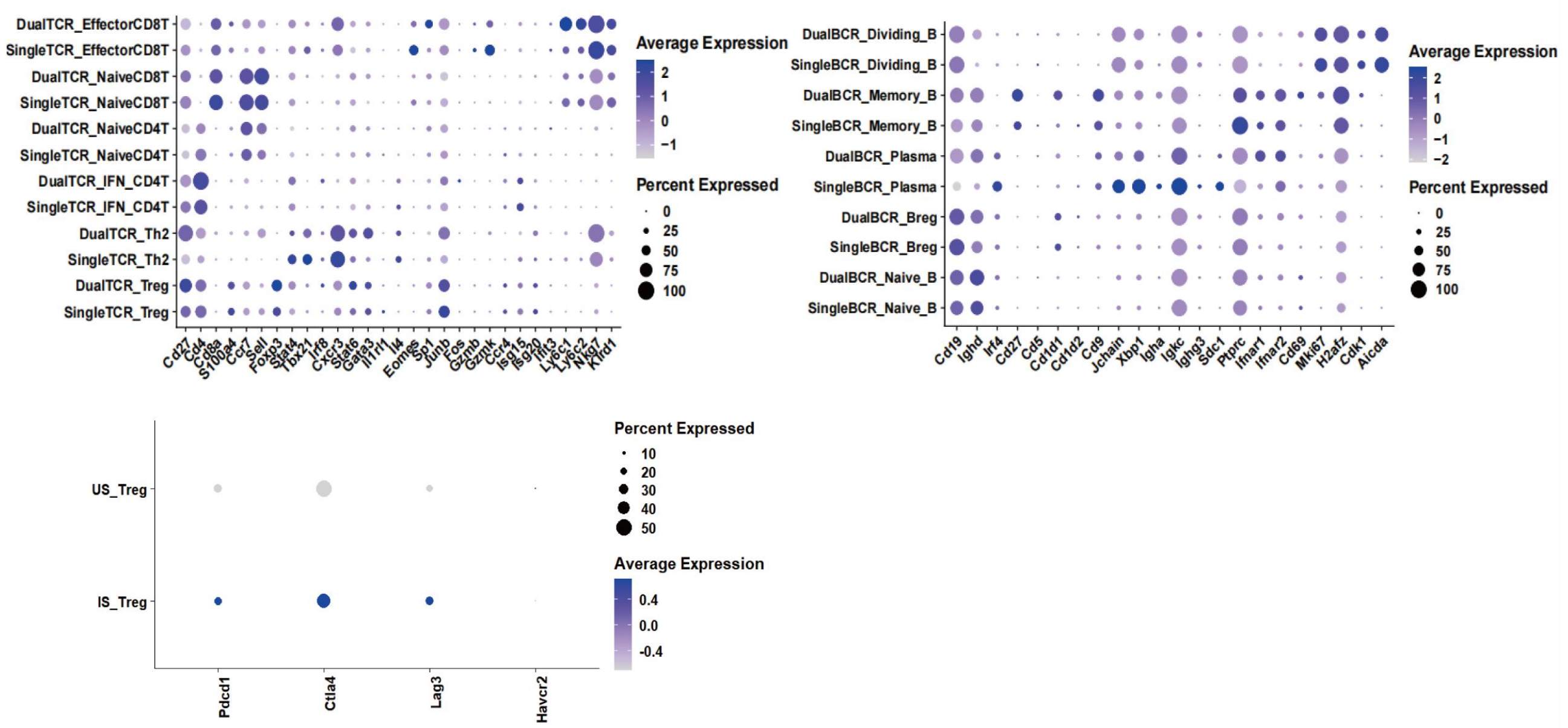

B

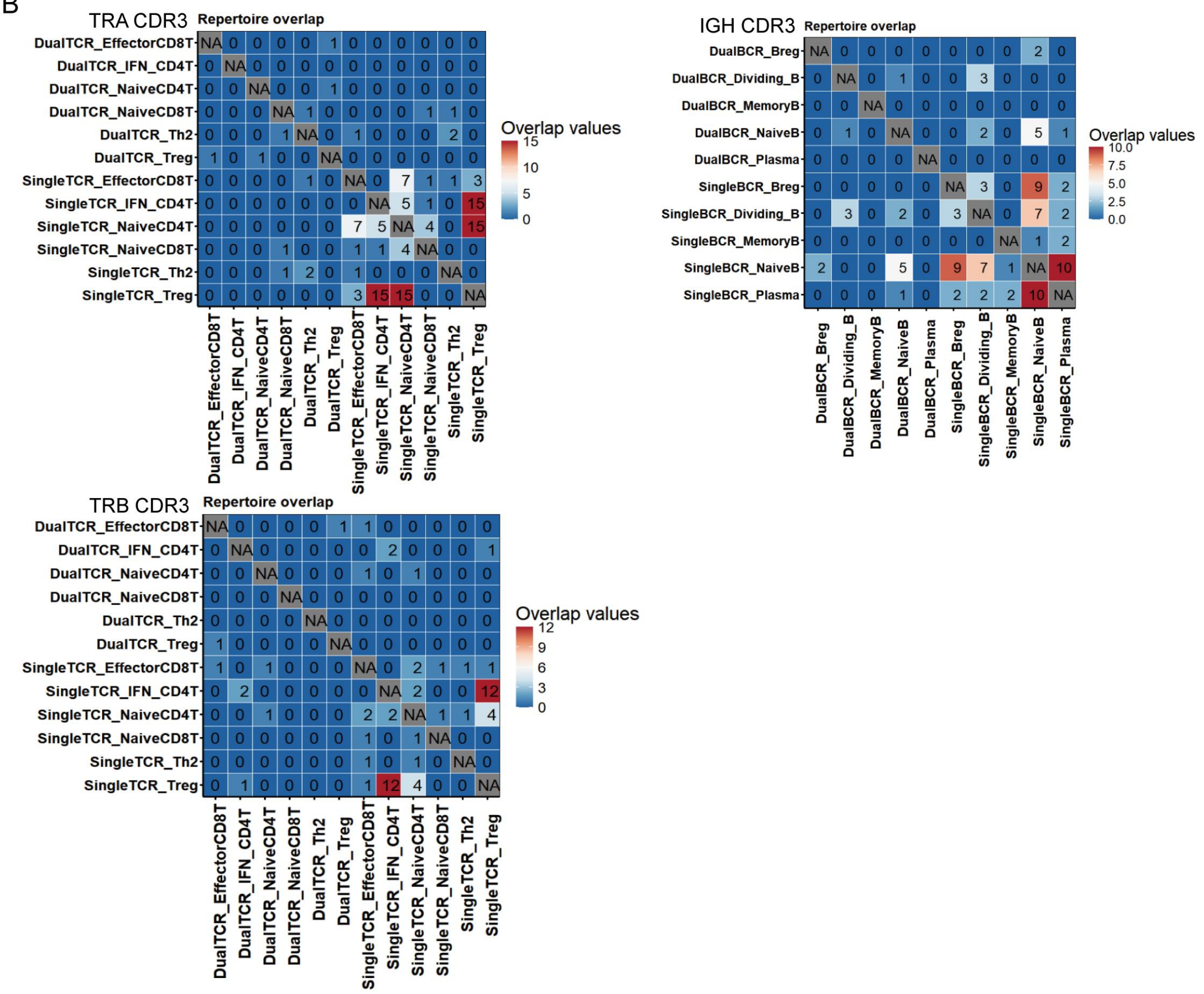

Supplementary Figure 3. Analysis of characteristic transcriptional genes and CDR3 overlaps in single TCR T, dual TCR T, single BCR B, and dual BCR B cell subsets of the IS group using scTCR+TCR+BCR-seq.

A. Analysis of characteristic genes and cytokines expression in single TCR T and dual TCR T cell subsets of the IS group; analysis of characteristic genes and cytokines expression in single BCR B and dual BCR B cell subsets of the IS group; analysis of dual TCR Treg cells expressing Pdcd1, Ctla4, Lag3, and Havcr2 in the IS and US groups.

B. Number of overlapping TRA CDR3 AA sequences in single TCR T and dual TCR T cell subsets of the IS group; number of overlapping TRB CDR3 AA sequences in single TCR T and dual TCR T cell subsets; number of overlapping IGH CDR3 AA sequences in single BCR B and dual BCR B cell subsets.

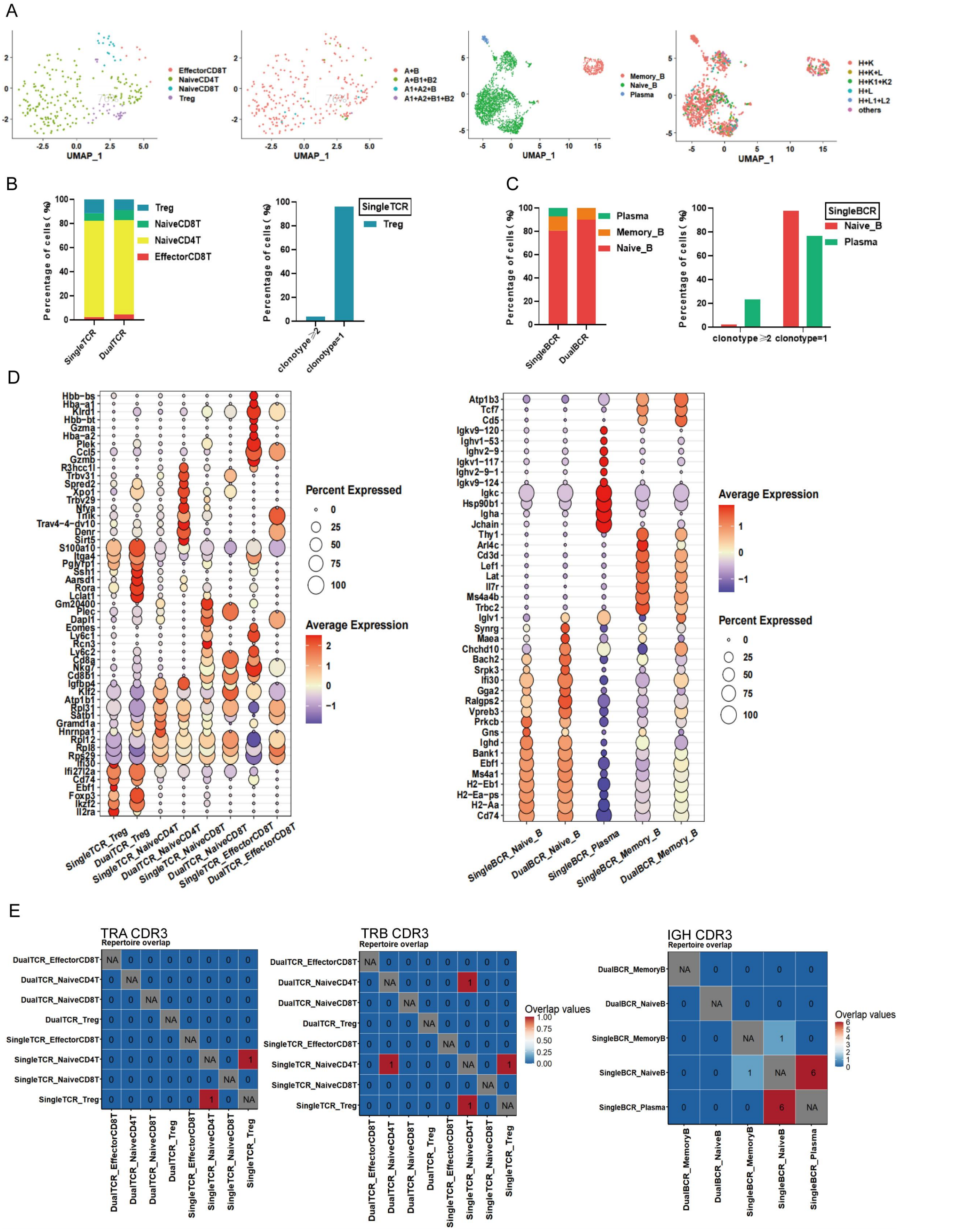

Supplementary Figure 4. Analysis of T and B cell subsets, clonality, mRNA expression, and CDR3 overlap in the UP group using scTCR+TCR+BCR-seq.

A. UMAP clustering maps of T cell and B cell subsets; UMAP clustering maps of TCR and BCR pairing types.

B. Analysis of the origin of single TCR T and dual TCR T cell subsets and their corresponding clonal proliferation.

C. Analysis of the origin of single BCR B and dual BCR B cell subsets and their corresponding clonal proliferation.

D. Comparison of the top 10 mRNA expressions in single TCR T and dual TCR T cells; comparison of the top 10 mRNA expressions in single BCR B and dual BCR B cells.

E. Number of overlapping TRA CDR3 AA sequences and TRB CDR3 AA sequences in single TCR T and dual TCR T cell subsets; number of overlapping IGH CDR3 AA sequences in single BCR B and dual BCR B cell subsets.

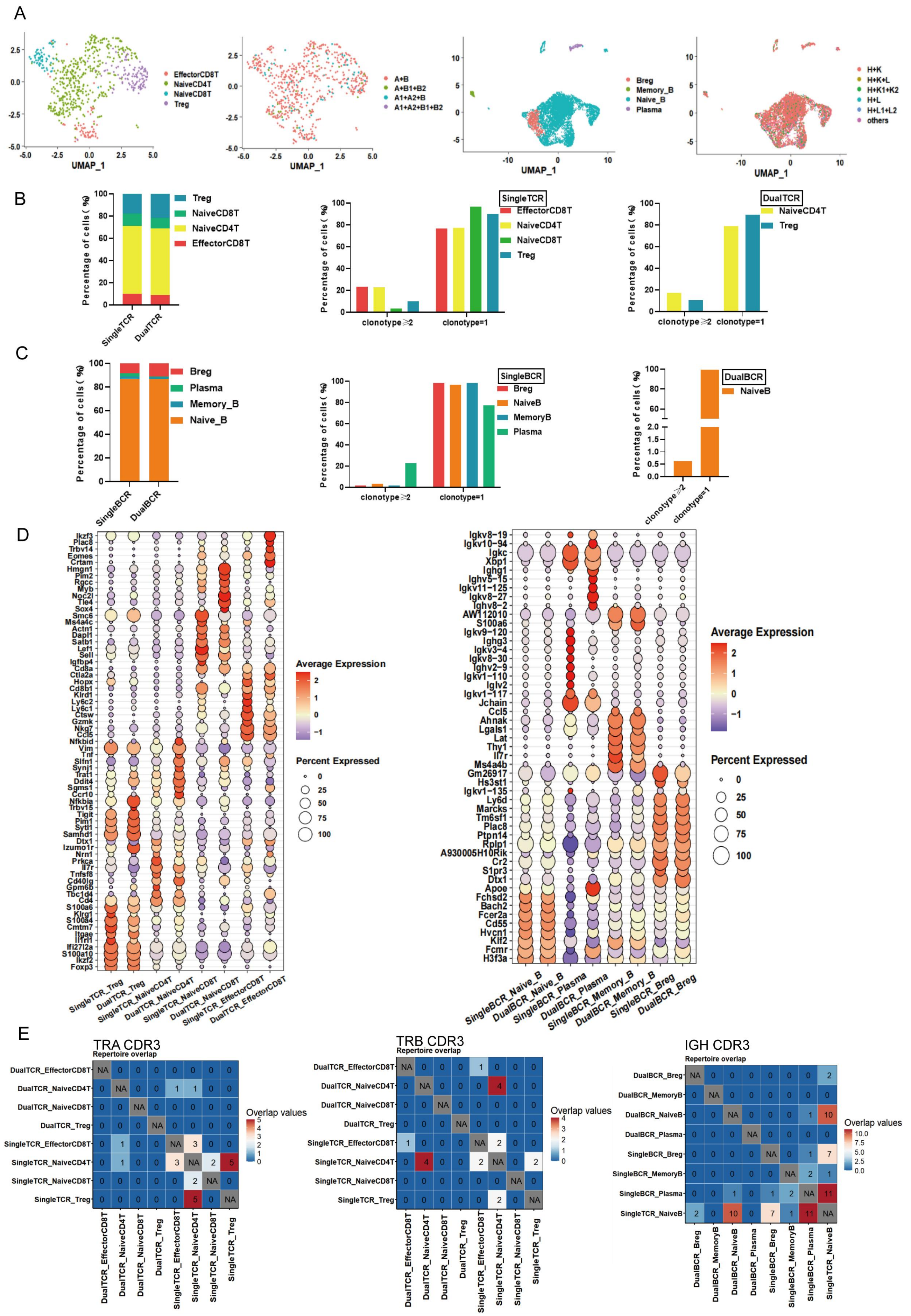

Supplementary Figure 5. Analysis of T and B cell subsets, clonality, mRNA expression, and CDR3 overlap in the

US group using scTCR+TCR+BCR-seq.

A. UMAP clustering maps of T cell and B cell subsets; UMAP clustering maps of TCR and BCR pairing types.

B. Analysis of the origin of single TCR T and dual TCR T cell subsets and their corresponding clonal proliferation.

C. Analysis of the origin of single BCR B and dual BCR B cell subsets and their corresponding clonal proliferation.

D. Comparison of the top 10 mRNA expressions in single TCR T and dual TCR T cells; comparison of the top 10 mRNA expressions in single BCR B and dual BCR B cells.

E. Number of overlapping TRA CDR3 AA sequences and TRB CDR3 AA sequences in single TCR T and dual TCR T cell subsets; number of overlapping IGH CDR3 AA sequences in single BCR B and dual BCR B cell subsets.

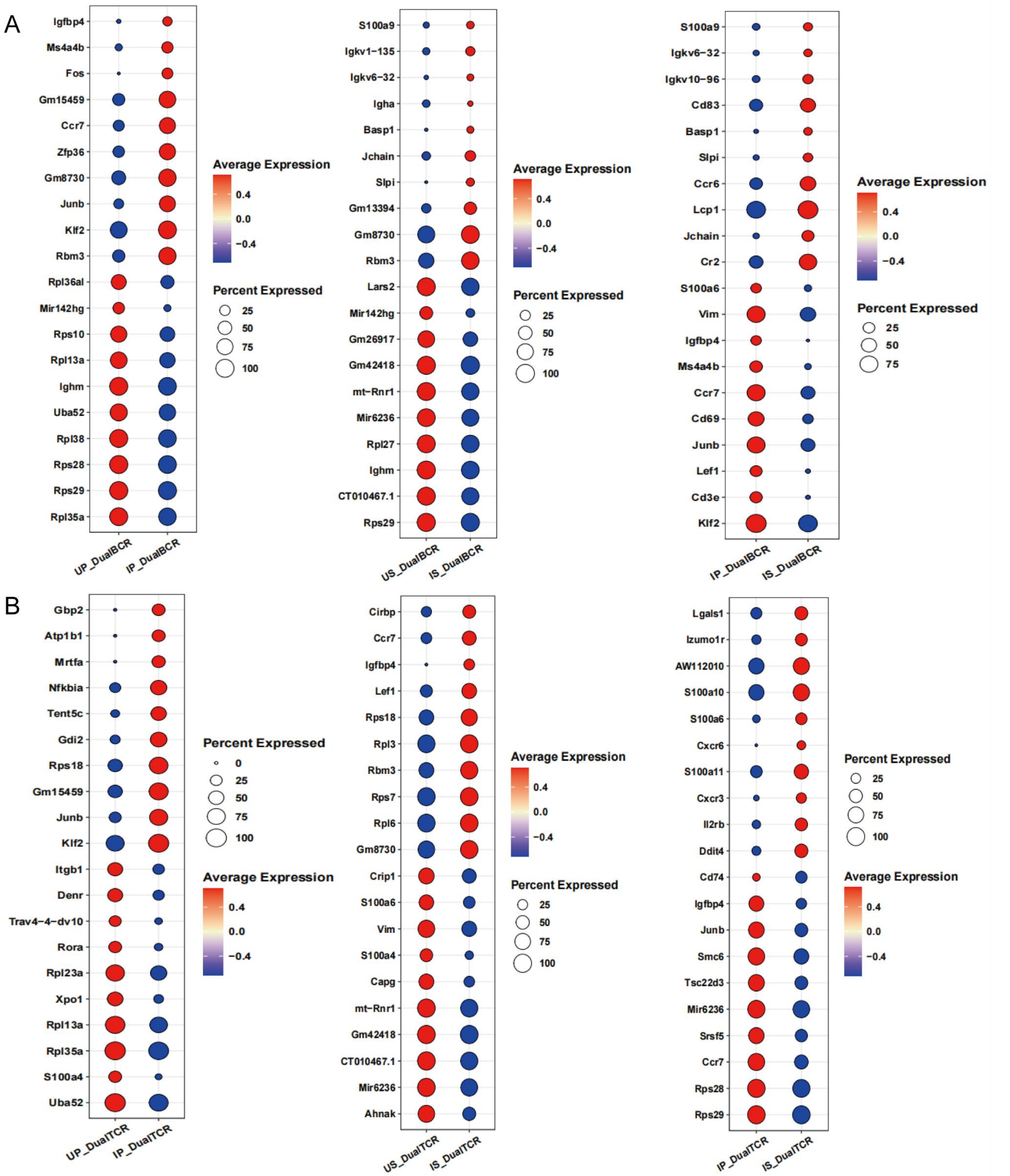

Supplementary Figure 6. Expression of the top 10 mRNAs in total dual TCR T cells and total double BCR B cells in four groups (IP, IS, UP, US) analyzed by scRNA+TCR+BCR-seq.

A, Comparison of the top 10 differentially expressed transcripts in double BCR B cells between groups (IP vs. UP, IS vs. US, IP vs. IS).

B, Comparison of the top 10 differentially expressed transcripts in dual TCR T cells between groups (IP vs. UP, IS vs. US, IP vs. IS).

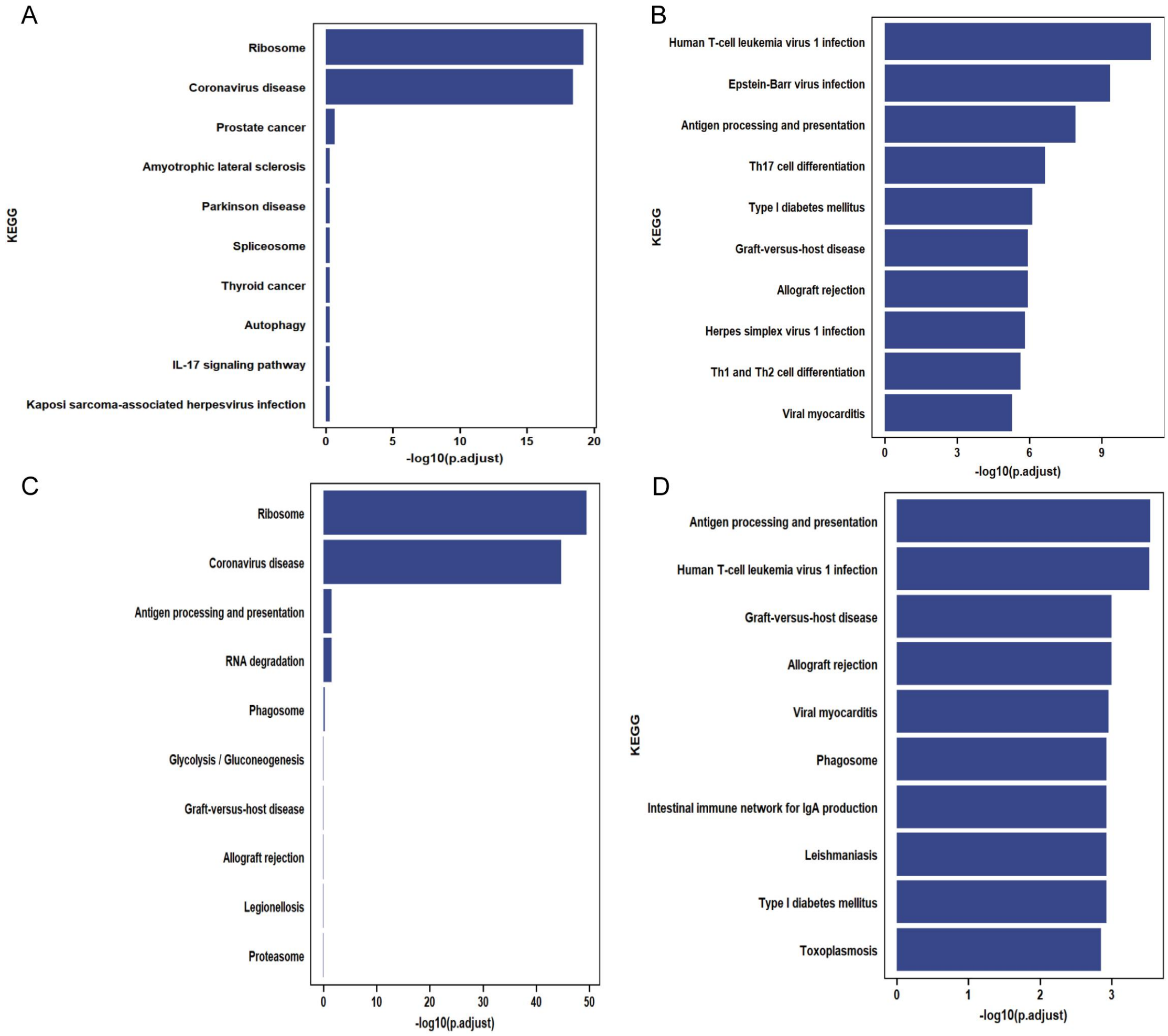

Supplementary Figure 7. Gene enrichment analysis of total dual TCR T cells and total dual BCR B cells in two groups (IP, IS) using scTCR+TCR+BCR-seq.

A. KEGG enrichment analysis of dual TCR T cells in peripheral blood samples from the infected group.

B. KEGG enrichment analysis of dual BCR B cells in peripheral blood samples from the infected group.

C. KEGG enrichment analysis of dual TCR T cells in splenic samples from the infected group.

D. KEGG enrichment analysis of dual BCR B cells in splenic samples from the infected group.

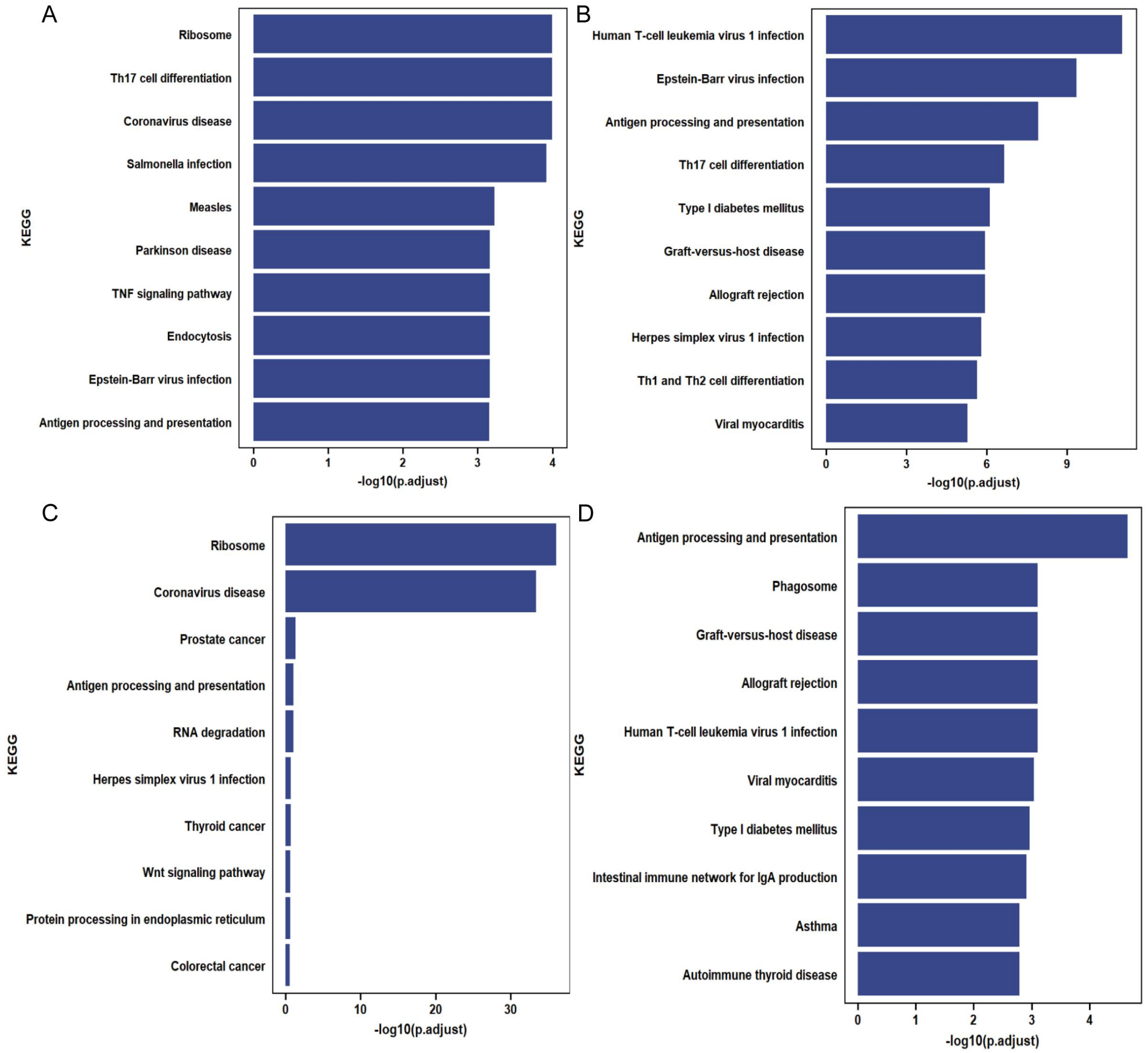

Supplementary Figure 8. Gene enrichment analysis of total single TCR T cells and total single BCR B cells in two groups (IP, IS) using scTCR+TCR+BCR-seq.

A. KEGG enrichment analysis of single TCR T cells in peripheral blood samples from the infected group.

B. KEGG enrichment analysis of single BCR B cells in peripheral blood samples from the infected group.

C. KEGG enrichment analysis of single TCR T cells in splenic samples from the infected group.

D. KEGG enrichment analysis of single BCR B cells in splenic samples from the infected group.
